# A systematic pan-cancer study demonstrates the oncogenic function of heterogeneous nuclear ribonucleoprotein C

**DOI:** 10.18632/aging.203981

**Published:** 2022-03-28

**Authors:** Chenxi Pan, Qian Wu, Nianjie Feng

**Affiliations:** 1 Key Laboratory of Fermentation Engineering (Ministry of Education), National “111” Center for Cellular Regulation and Molecular Pharmaceutics, Hubei Key Laboratory of Industrial Microbiology, Hubei Research Center of Food Fermentation Engineering and Technology, Hubei University of Technology, Wuhan 430068, China; 2 The Second Affiliated Hospital of Dalian Medical University, Dalian 116027, China

**Keywords:** HNRNPC, prognosis, phosphorylation, pan-cancer, immune infiltration

## Abstract

Although complex links between heterogeneous nuclear ribonucleoprotein C (HNRNPC) and numerous types of cancer have been shown in both cell and animal models, a comprehensive pan-cancer investigation on the features and activities of HNRNPC is still lacking. Based on the Cancer Genome Atlas and Gene Expression Omnibus datasets, we investigated the possible oncogenic effects of HNRNPC in thirty-three cancers. HNRNPC expression was detected in the majority of cancers, and its expression level was shown to be significantly linked with cancer patient prognosis. HNRNPC increased the phosphorylation of S220, which was detected in various cancers, including ovarian cancer and colon cancer. HNRNPC expression was also shown to be related to cancer-associated cell infiltration, most notably in uveal melanoma, testicular germ cell tumors, and thymoma. Additionally, the signaling pathway for vascular endothelial growth factors and RNA transport were implicated in HNRNPC's functioning processes. In short, HNRNPC may further influence cancer progression through gene mutation, protein phosphorylation, cancer associated fibroblasts infiltration and related molecular pathways. This work was intended to provide a relatively thorough knowledge of the oncogenic activities of HNRNPC across a variety of tumor types by performing a systematic pan-cancer investigation.

## INTRODUCTION

Malignant tumors are generally acknowledged as a main cause of death and a significant impediment to patients' quality of life in the majority of countries worldwide [1]. Despite recent advancements in neoadjuvant chemoradiation, targeted therapy, and immunotherapy, the overall survival rates continue to be low [2]. As a result, it is critical to discover novel tumor indicators to accomplish early identification, diagnosis, and therapy.

Heterogeneous nuclear ribonucleoproteins C (HNRNPCs) are members of the HNRNP family which are represented by two subtypes in humans (C1 and C2) [3, 4]. HNRNPC is well-known for its activity in alternative splicing regulation as an RNA binding protein [5]. Alternative splicing regulators are required for RNA maturation, stabilization, translocation, and posttranscriptional modification [6]. HNRNPC is required for the expression of genes and the proper functioning of biological processes [7–9]. Numerous researches have been conducted in recent years to examine the association between HRNNPC and cancer, which is one of the significant outcomes of aberrant gene expression and biological processes. HNRNPC expression has been reported to be abnormal in a number of malignancies, including prostate cancer [10], breast cancer (BRCA) [11, 12], pancreatic ductal adenocarcinoma [13], lung adenocarcinoma (LUAD) [14], glioblastoma [15], oral squamous cell carcinoma, and hepatocellular carcinoma [16]. Additional research revealed that HNRNPC was involved in the processing of cancer-related genes, including BRCA1/2, uPAR, MALAT, PDCD4, and cMyc [17–20]. HNRNPC is certainly related to several types of cancer. However, the differential expression of HNRNPC has an uncertain effect on the prognosis of many malignancies, and there is no universally accepted criterion for determining the significance of HNRNPC in distinct cancers. The purpose of this study is to determine the expression and effect of HNRNPC in pan-cancer and to determine whether HNRNPC can be used as a universal cancer marker.

By examining numerous profiles of a large number of human cancers, the pan-cancer project will evaluate the similarities and variations in both genetic and cellular changes across distinct tumor types [21]. On one hand, pan-cancer studies can reflect the cross-tumor similarity reflected by different levels of molecular characteristics. On the other hand, pan-cancer studies have the advantage of having a large sample size. Pan-cancer studies have demonstrated their advantages in finding novel common biomarkers. In this study, gene and protein expression, survival status, genetic alteration, phosphorylation, immune infiltration, and molecular pathways were performed for pan-cancer analysis of HNRNPC. The findings of this study are expected to offer a greater insight on the possible molecular mechanism through which HNRNPC contributes to the development or clinical prognosis of various malignancies.

## MATERIALS AND METHODS

### Gene expression analysis

We examined the Cancer Genome Atlas (TCGA) database using the TIMER2 (tumor immune estimation resource, version 2) online resource's “Gene DE” module for changes in HNRNPC expression between the tumor and its nearby normal tissues for distinct malignancies or tumor subtypes. To generate box plots illustrating the differential expression among these tumor tissues and their matching normal tissues in the GTEx (Genotype-Tissue Expression) database, we utilized the GEPIA2 (Gene Expression Profiling Interactive Analysis, version 2) web server's “Expression analysis-Box Plots” module [22] with the following settings: P-value threshold = 0.01, log2FC (fold change) = 1. Additionally, we used HEPIA2's “Clinical Stage Plot” module to produce violin plots of HNRNPC expression in each of the TCGA tumors' distinct clinical stages, which we then compared with one another (stage I, stage II, stage III, and stage IV). Furthermore, we used log2 [TPM (Transcripts per million) + 1] transformed expression data from the TPM database as the input for the box and the violin graphs.

In this study, we used the University of Alabama Cancer Database (UALCAN) portal, an interactive online resource for reviewing cancer Omics data [23]. The Clinical proteomic tumor analysis consortium (CPTAC) dataset was also analyzed for protein expression by using the UALCAN portal. Our research team evaluated the levels of HNRNPC (NP 001070910.1) total protein and phosphoprotein expression in primary tumor and normal tissues after entering the term “HNRNPC” into the search box. From the available datasets, BRCA, ovarian cancer, colon cancer, clear cell renal cell carcinoma (ccRCC), uterine corpus endometrial carcinoma (UCEC), LUAD, and pediatric brain cancer were selected for further investigation and analysis.

### Staining via immunohistochemistry

Immunohistochemical images of HNRNPC protein expression studies were performed in normal and 10 tumor tissues from the HPA, including liver cancer, lung cancer, BRCA, prostate cancer, and colon cancer, to examine variations in HNRNPC expression at the protein level. HPA51075 was the antibody used for IHC. The number of tumor samples with IHC was 10–12. Antibody staining in cancer types in contemporary human tissue is recorded as not observed, low, medium, or high in the HPA dataset. The intensity of the staining and the proportion of stained cells are used to calculate this score.

### Analyses of survival prognoses

Researchers used the “Survival Map” module of the GEPIA2 software to produce the overall survival (OS) and disease-free survival (DFS) significance maps of HNRNPC over all TCGA tumors [22]. Using cutoff values of 50% for high expression and 50% for low expression, respectively, it was possible to distinguish between high and low expression groups. GEPIA2's “Survival Analysis” module was used to create survival plots, which together with the log-rank test were subsequently used to test our hypothesis.

### Analysis of genetic variations

When we logged onto the cBioPortal website [24, 25], we were able to quickly obtain information on HNRNPC's genetic alteration features by selecting “TCGA pan-cancer Atlas Studies” from the “Quick select” area. This module summarized the mutation frequency, mutation type, and copy number alteration (CNA) statistics across all TCGA tumors in a single report. The “Mutations” module displays information on HNRNPC mutations in the form of a schematic depiction or as a three-dimensional (3D) representation of the protein structure. Additionally, we will use the “Comparison” module to investigate whether there are any differences in OS, DFS, Progression-free survival (PFS), and Disease-specific survival between TCGA cancer patients who have and do not have HNRNPC genetic variations. Additionally, log-rank P-values were used to generate Kaplan-Meier plots, which were then graphically represented.

### Immune infiltration analysis

We evaluated the connection between HNRNPC expression and immune infiltrates in all of the TCGA tumor samples by using the “Immune-Gene” module of the TIMER2 web server and its “Immune-Gene” module. Fibroblast cells and immune cells that have been linked to cancer were selected for the study. This was determined by the presence of immune infiltration utilizing a variety of algorithms, including the TIMER, CIBERSORT, CIBERSORT-ABS, QUANTISEQ, XCELL, MCPCOUNTER, and EPIC, among others. We were able to calculate the P-values and the partial correlation (Cor) values with the purity-adjusted Spearman's rank correlation test. Information was illustrated using a heatmap and a scatter plot, respectively.

### Gene enrichment study for HNRNPC-related genes

Finding matches in the STRING database successfully required us to search for a single protein name (“HNRNPC”) and a single organism (“Homo sapiens”). Because of this, we defined the following basic parameters: the lowest required interaction score [“Low confidence (0.150),” the meaning of network edges (“evidence”), the maximum number of interactors to display (“no more than 50 interactors” in the first shell), and active interaction sources (“experiments”). Furthermore, HNRNPC-binding proteins, which had already been found and experimentally extracted, were also identified and isolated.

Based on the datasets of all TCGA pan-cancer Atlas studies, we used the “Coexpression” module of the cBioPortal web to determine the top 200 HNRNPC correlated targeting genes. With the use of GEPIA2's “correlation analysis” module, we were able to conduct pairwise gene Pearson correlation research on HNRNPC and selected genes as well. The log2 TPM was used to generate the dot plot. The correlation coefficient (R) as well as the P-value were shown on the screen. Additionally, we utilized TIMER2's “Gene Corr” module to create heatmap data for the chosen genes, which contained the partial correlation (Cor) and the P-value for the purity-adjusted Spearman's rank correlation test. We also used the TIMER2 “Gene Corr” module to generate heatmap data for the selected genes. We also used Jvenn, an interactive Venn diagram viewer [26] that allows us to perform an intersection analysis, to compare the HNRNPC-binding and interacting genes. In addition, we combined the two data sets to conduct a KEGG pathway analysis using the combined data (Kyoto encyclopedia of genes and genomes). We submitted the gene lists to KOBAS (KEGG Orthology Based Annotation System) with the required identifier (“Gene list enrichment”) and species (“Homo sapiens”) settings, and we obtained the functional annotation Visualization as a result. As part of the GO (Gene ontology) enrichment study, we also used Metascape.

## RESULTS

### HNRNPC expression in pan-cancer

**Figure 1:**
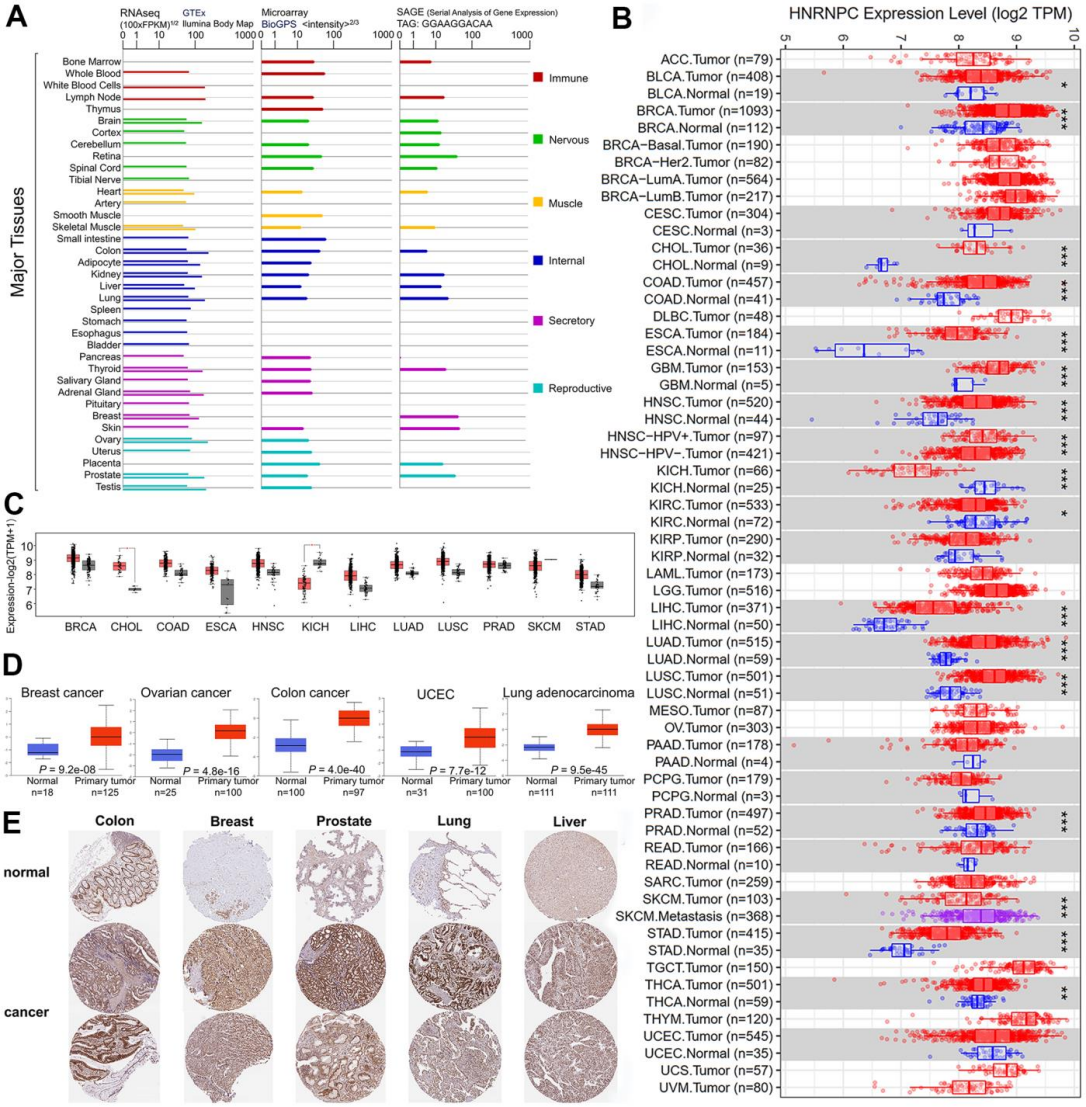
**HNRNPC expression in different tumor type.** (**A**) The expression levels of the HNRNPC gene varies according to tumor type. in GTEx, Illumina, BioGPS, and SAGE. (**B**) TIMER2 was used to determine the amount of expression of the HNRNPC gene in various malignancies or particular cancer subtypes. (**C**) The TCGA project provided box plot data for pan-cancer, as well as normal tissues from the GTEx database as controls. (**D**) The expression level of HNRNPC total protein was determined in normal and primary tissues using the CPTAC dataset. (**E**) HPA-induced expression of the HNRNPC protein in tumor and tumor-adjacent normal tissues. * P < 0.05; ** P < 0.01; *** P < 0.001.

In order to explore the relationship between HNRNPC and various cancers, we first examined whether there were differences in the expression levels of RNA and protein of HNRNPC. We investigated the oncogenic potential of HNRNPC (NC 000014.9 for DNA, NM 004500.4 for mRNA, and NP 004491.2 for protein) by evaluating its expression pattern in a variety of cells and nontumor tissues. According to GTEx, Illumina, BioGPS, and SAGE datasets, HNRNPC is expressed in all observed tissues (all consensus normalized expression levels >1) and has a low tissue selectivity for RNA. Additionally, substantial levels of HNRNPC were detected in the breast, lymph nodes, and ovary (Figure 1A). The expression status of HNRNPC was evaluated using the TIMER2 technique across the TCGA's diverse cancer types. The level of HNRNPC expression in BRCA, Cholangiocarcinoma (CHOL), Colon adenocarcinoma, Esophageal carcinoma (ESCA), Glioblastoma multiforme (GBM), Head and Neck squamous cell carcinoma (HNSC), Kidney chromophobe (KICH), and Liver hepatocellular carcinoma (LIHC) was also assessed (Figure 1B). To further validate the expression of HNRNPC RNA levels in pan-cancer, we checked the results of GTEx database again. The difference in HNRNPC expression between normal and tumor tissues (CHOL and KICH) was further analyzed using the GTEx dataset (Figure 1C, P < 0.05). Combined with TCGA database and GTEx database, HNRNPC is expressed significantly differently in CHOL and KICH. The CPTAC dataset revealed that the HNRNPC total protein expression was significantly greater in primary BRCA, ovarian cancer, colon cancer, UCEC, and LUAD tissues than in normal tissues (Figure 1D). Consequently, the IHC results from the HPA were processed and compared to the TCGA-provided HNRNPC gene expression information to evaluate HNRNPC protein expression (Figure 1E). The results of the data analysis from the two databases were similar, and the HPA database filled in gaps in the TCGA data pertaining to HNRNPC expression in several normal tissues.

**Figure 2:**
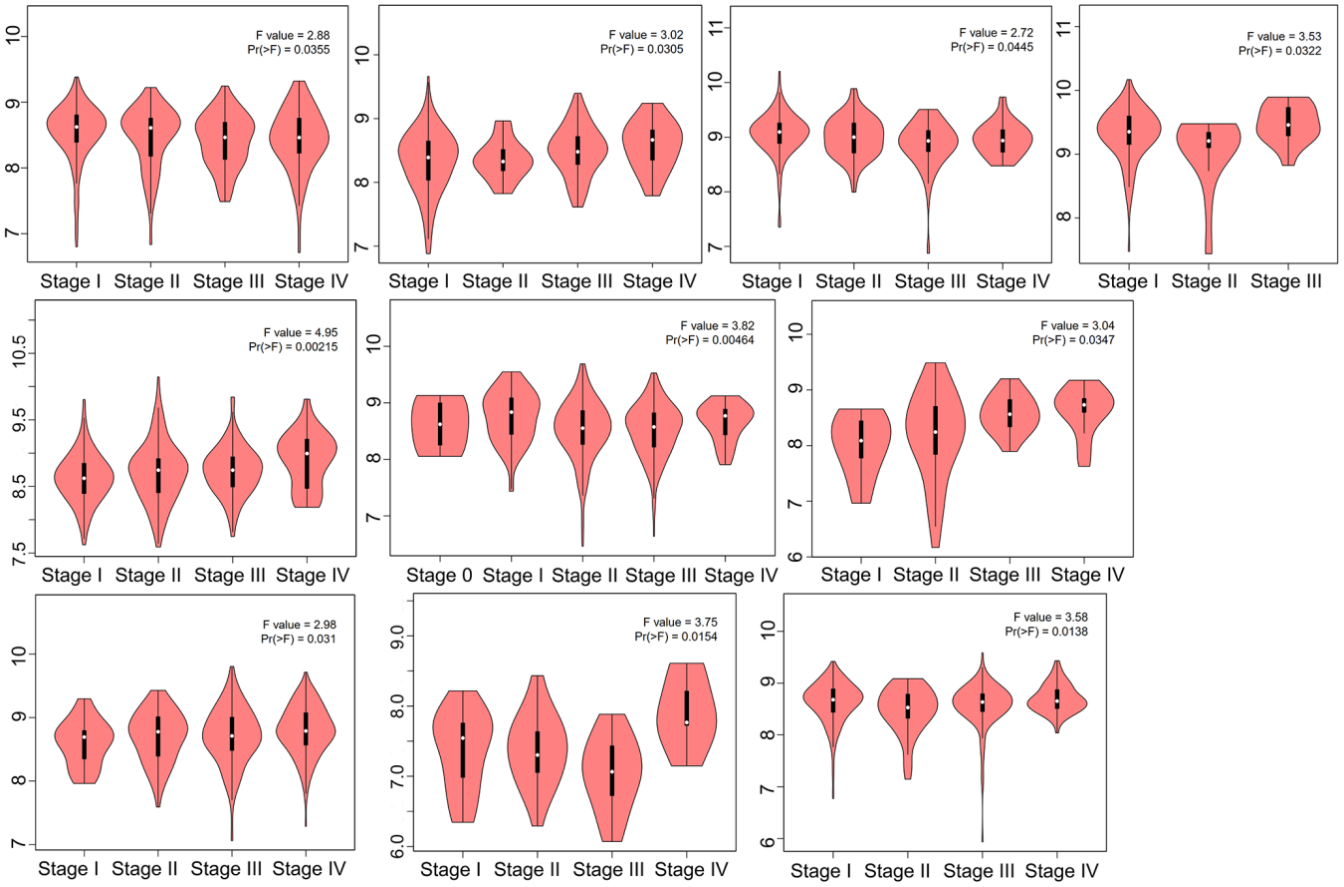
**HNRNPC expression in different clinical stage.** TCGA data were used to examine the expression level of the HNRNPC gene during the major pathogenic phases. For log-scale, Log2 (TPM+1) was used.

At the RNA level and protein level, HNRNPC showed significant differences between tumor tissue and normal tissue. These results support the possibility of HNRNPC as a carcinogen in many aspects. It will lay a foundation for further research on the carcinogenic effect of HNRNPC. The HEPIA2 module “Pathological Stage Plot” was used to examine the link between HNRNPC expression and the pathological stages of various malignancies, such as adrenocortical carcinoma (ACC), CESC, HNSC, KICH, kidney renal clear cell carcinoma (KIRC), kidney renal papillary cell carcinoma (KIRP), LUAD, skin cutaneous melanoma (SKCM), testicular germ cell tumors (TGCT), and thyroid carcinoma (Figure 2). The above results suggest that HNRNPC may contribute to the development and progression of cancer by influencing the progression of tumor staging.

### Relationship between prognosis and HNRNPC expression

**Figure 3:**
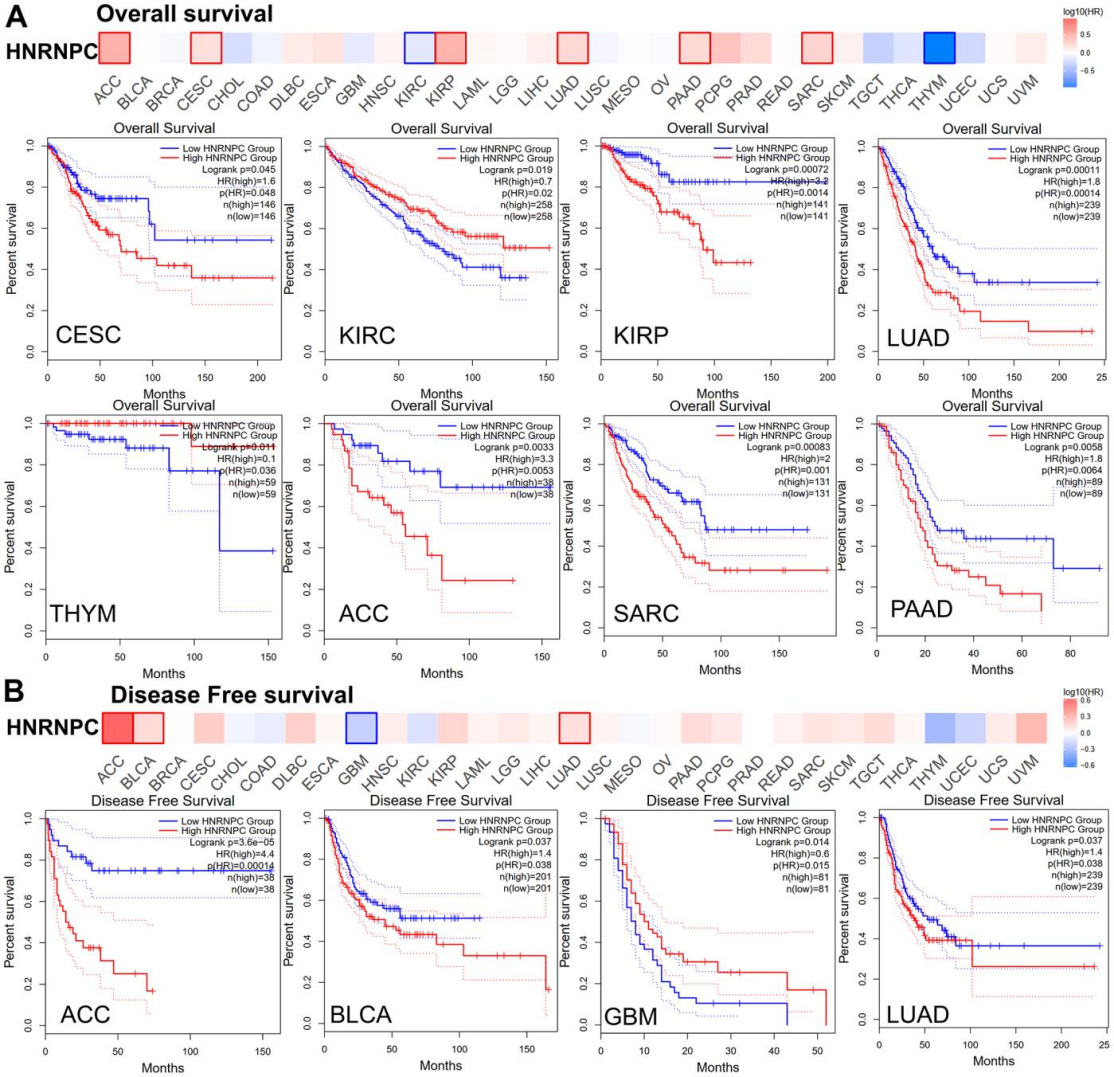
**Relationship between HNRNPC gene expression and survival prognosis of cancers in TCGA.** (**A**) overall survival and (**B**) disease-free survival.

To further explore whether the expression of HNRNPC will affect the survival of cancer patients, we analyzed the prognosis of OS and DFS in patients with high and low HNRNPC expression group. According to their levels of HNRNPC expression, cancer patients were distinguished into high- and low expression groups. The connection between HNRNPC expression and prognosis in patients with various types of malignancies was subsequently examined using the TCGA and GEO datasets. The significantly increased HNRNPC was associated with a poor OS prognosis in the TCGA project for LUAD (P = 0.00011), CESC (P = 0.045), KIPR (P = 0.00072), ACC (P = 0.0033), Sarcoma (SARC) (P = 0.00083), and Pancreatic adenocarcinoma (PAAD) (P = 0.0058) (Figure 3A). The DFS study revealed a connection between high HNRNPC expression and a poor prognosis for LUAD (P = 0.037), ACC (P = 3.6e−05), and BLCA (P = 0.037) patients from the TCGA (Figure 3B). Furthermore, reduced HNRNPC expression was associated with a poor DFS prognosis for GBM (P = 0.014) and a poor OS prognosis for KIRC (P = 0.019) and THYM (P = 0.011).

### Relationship between gene mutation characteristics and HNRNPC expression

**Figure 4:**
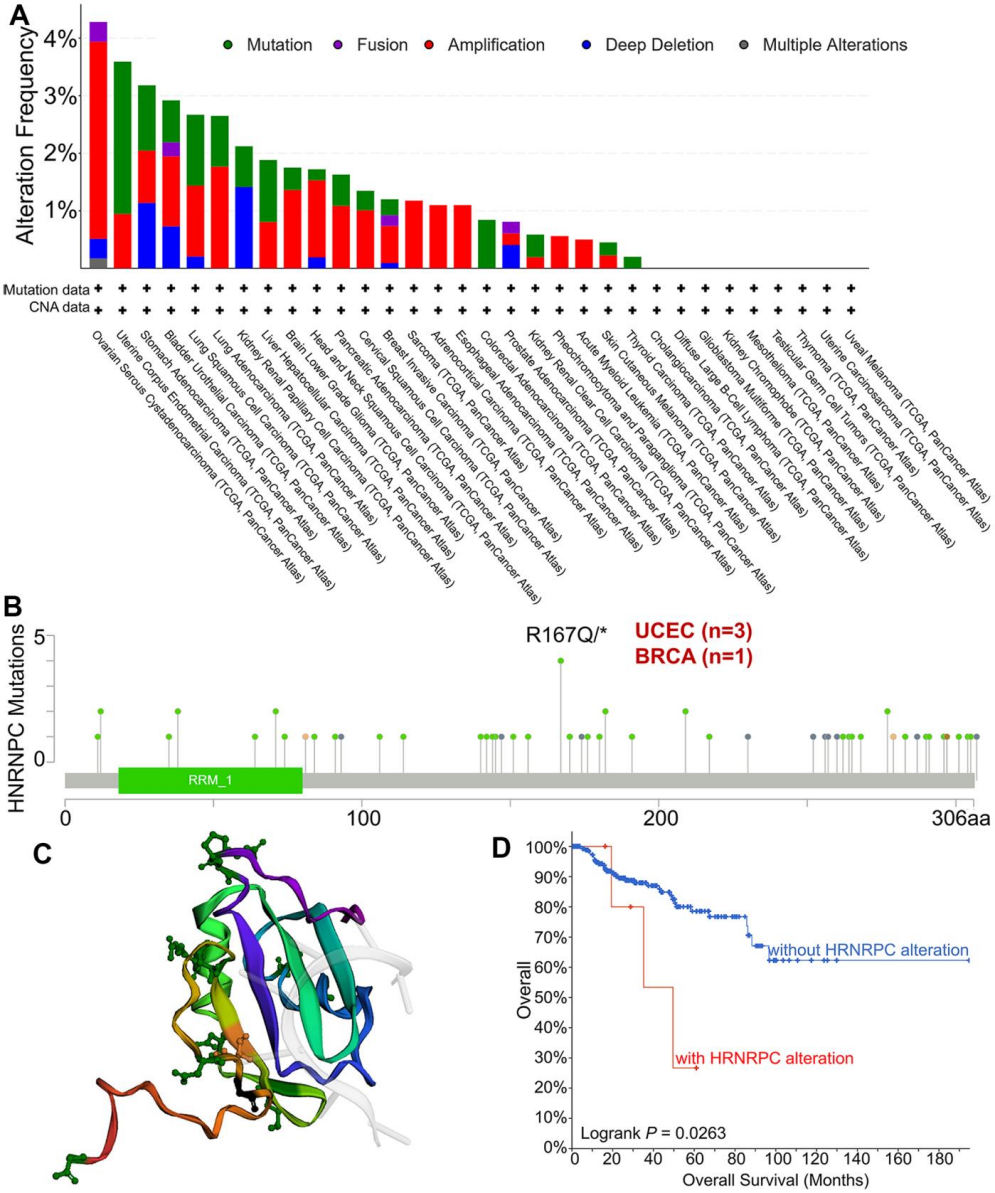
**Mutation types and mutation sites of HNRNPC in TCGA tumors.** (**A**) Mutation frequency and mutation type of HNRNPC in pan-cancer. (**B**) The mutation site of HNRNPC and the number of related cases at this mutation site. (**C**) The three-dimensional structure of HNRNPC. (**D**) the possible link between mutation status and overall survival in Kidney Renal Papillary Cell Carcinoma (P<0.05).

To investigate whether HNRNPC causes cancer through gene mutation, we analyzed the mutation sites and types of HNRNPC in pan-cancer. The genetic status of HNRNPC was determined in several tumor samples from the TCGA cohorts. The highest frequency of HNRNPC change was 4.28 %, occurring in Ovarian Serous Cystadenocarcinoma with “mutation” as the major type (Figure 4A). Additionally, the “amplification” kind of CNA had a much higher modification frequency of 3.42%. Additionally, it was noteworthy that all SARC, ACC, and ESCA patients with a genetic change (>1% frequency) showed HNRNPC CNA. Figure 4B depicts the types, sites, and case numbers of the HNRNPC genetic mutation. The primary form of the genetic mutation was a missense mutation in HNRNPC. Three cases of UCEC and one case of BRCA, respectively, had an R167Q/* mutation in the Tudor domain. R167Q/* mutation is capable of causing a missense mutation in the HNRNPC gene, converting R (Arginine) to Q (Glutamine) at the 167 locations on the HNRNPC protein, and resulting in a missense HNRNPC protein. Regrettably, the R167 mutation cannot be mapped onto the 3D structure of HNRNPC (Figure 4C). Then, we further explored the effect of HNRNPC mutations on the prognosis of pan-cancer. The possible relationship between HNRNPC genetic variants and clinical survival outcomes in patients with various types of cancer was investigated. In comparison to patients without the HNRNRPC mutation, KIPR demonstrated a worse prognosis (P = 0.0263) (Figure 4D).

### Relationship between protein phosphorylation and HNRNPC expression

**Figure 5:**
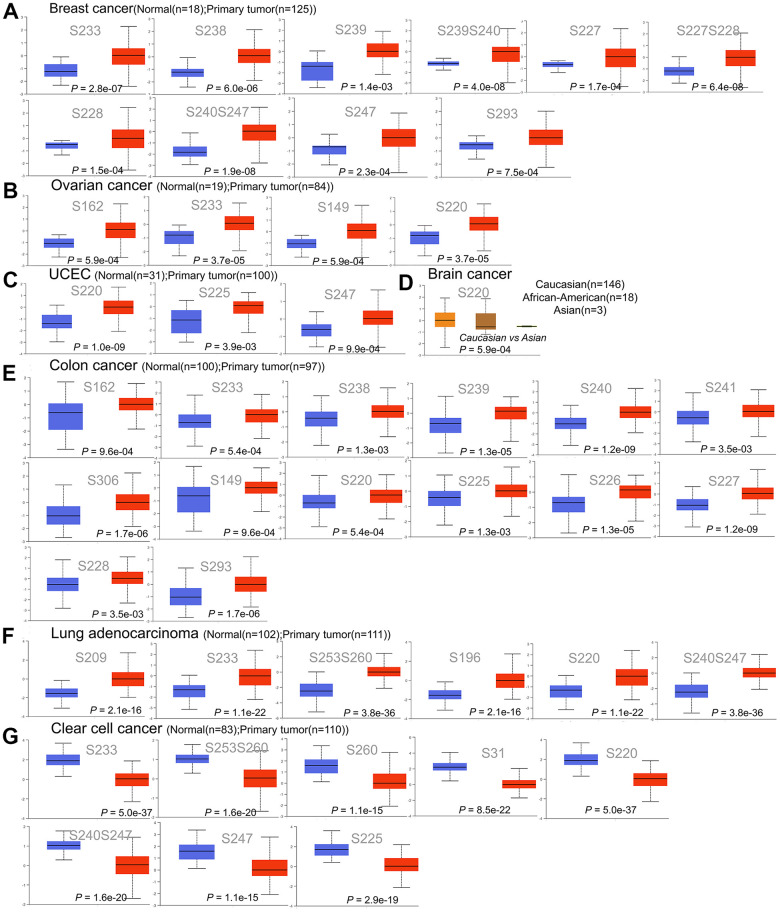
**Phosphorylation of the HNRNPC protein in a variety of cancers.** The UALCAN was used to compare the expression levels of HNRNPC phosphoprotein in normal and primary tissue using the CPTAC dataset. (**A**) Phosphorylation sites of HNRNPC proteins in breast cancer, including S233, S238, S239, S239S240, S227, S277S228, S228, S240S247, S247 and S293. (**B**) Phosphorylation sites of HNRNPC proteins in ovarian cancer, including S162, S233, S149 and S220. (**C**) Phosphorylation sites of HNRNPC proteins in UCEC, including S220, S225 and S247. (**D**) Phosphorylation sites of HNRNPC proteins in brain cancer, including S220. (**E**) Phosphorylation sites of HNRNPC proteins in colon cancer, including S162, S233, S238, S239, S240 and S241. (**F**) Phosphorylation sites of HNRNPC proteins in Lung adenocarcinoma, including S209, S233, S253S260, S196, S230 and S240S247. (**G**) Phosphorylation sites of HNRNPC proteins in clear cell cancer, including S233, S253S260, S260, S31, S220, S240S247, S247 and S255. Blue box plot: normal tissue; red box plot: tumor tissue; orange box plot: Caucasian; brown box plot: African-American; green box plot: Asian.

To clarify whether HNRNPC affects cancer through protein phosphorylation, we analyzed the existing protein phosphorylation database. By analyzing existing protein phosphorylation sites, we wanted to identify the most common mutation sites for HNRNPC. We compared the phosphorylation levels of HNRNPC in both normal and primary tumor tissues. Six distinct types of cancer were investigated using the CPTAC dataset (BRCA, colon cancer, ccRCC, LUAD, ovarian cancer, and UCEC). Figure 5 illustrates the HNRNPC phosphorylation sites and their substantial variations. In primary tumor tissues, the S220 of HNRNPC was more phosphorylated than in normal tissues (P < 0.05). This in turn raised the possibility that S220 phosphorylation plays a role in tumorigenesis.

### Relationship between cancer associated fibroblasts and HNRNPC expression

**Figure 6:**
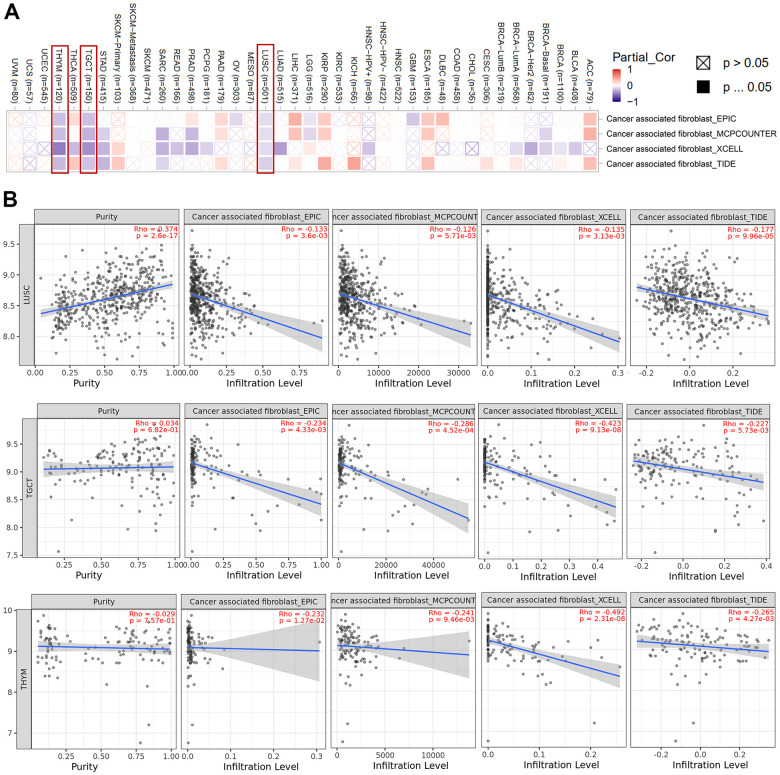
**Cancer-associated fibroblasts associated with HNRNPC expression.** (**A**) Heat map showing cancer-associated fibroblasts associated with HNRNPC expression in pan-cancer. (**B**) HNRNPC expression and cancer-associated fibroblasts estimation value scatter plots in LUSC, TGCT and THYM.

Tumor-infiltrating immune cells have been shown to play an important role in the origin, development, and metastasis of cancer. Here, we explored the effect of HNRNPC on key components of the tumor microenvironment. We employed the TIMER, CIBERSORT, CIBERSORT-ABS, QUANTISEQ, XCELL, MCPCOUNTER, and EPIC algorithms to investigate the relationship between immune cell infiltration and HNRNPC gene expression in a range of TCGA cancer types. It has been shown that cancer-associated fibroblasts in the tumor microenvironment stroma have an influential role on the activity of various tumor-infiltrating immune cells. We checked the effect of HNRNPC on cancer-associated fibroblasts. A statistically significant negative relationship between HNRNPC expression and the estimated infiltration value of cancer-associated fibroblasts was identified in the TCGA tumors LUSC, TGCT, and THYM (Figure 6A). Scatter plots assessing the possible correlations among cancers in the TCGA are shown in Figure 6B using the EPIC algorithm. The degree of HNRNPC expression in LUSC was inversely correlated with the level of cancer-associated fibroblast infiltration (Cor = −0.133, P = 3.6e−03).

### Relationship between molecular pathways and HNRNPC expression

**Figure 7:**
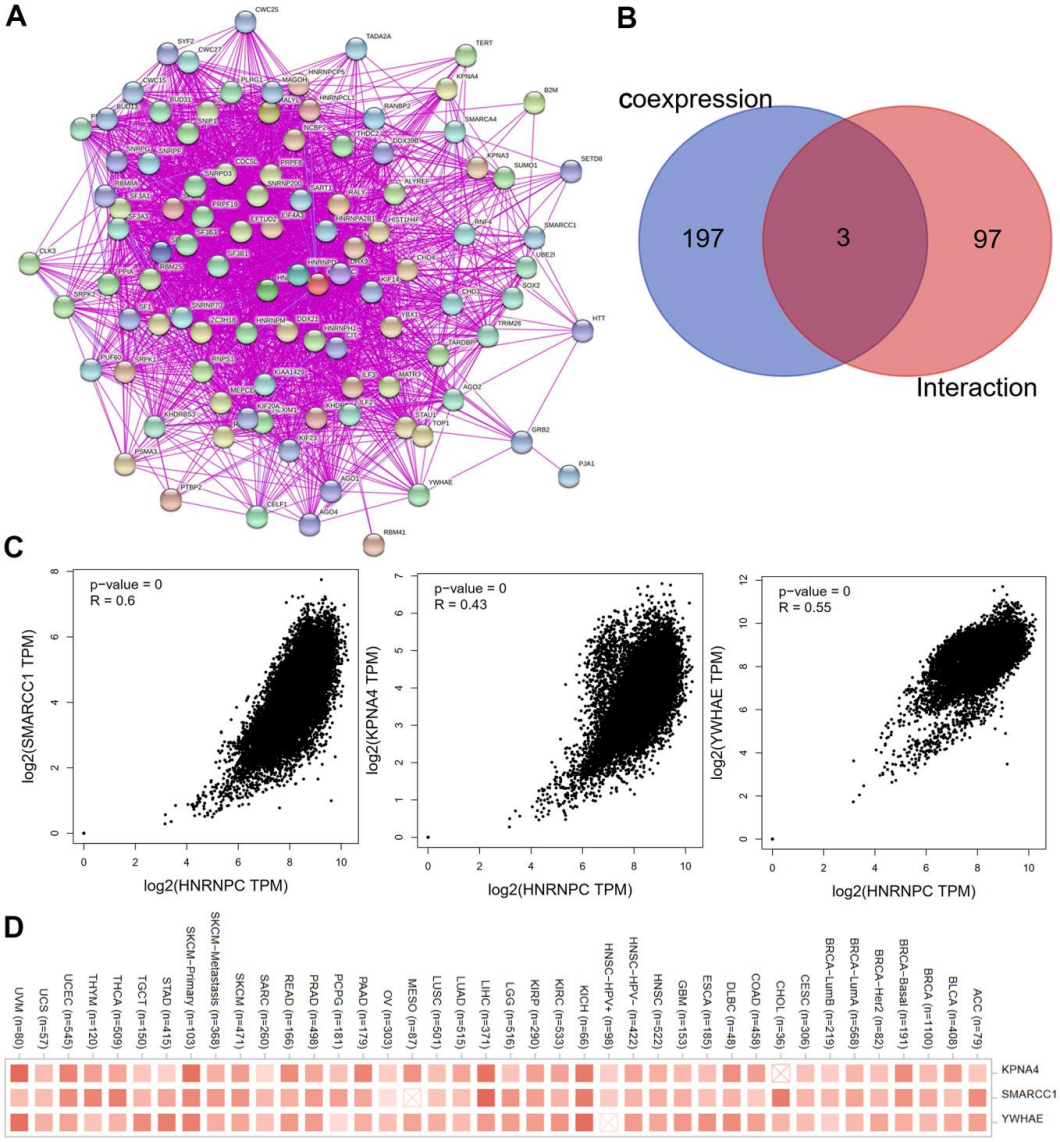
**Screening for HNRNPC-related genes.** (**A**) The interaction network of the first hundred experimentally determined HNRNPC-binding proteins by STRING. (**B**) Venn diagram was used to look at the intersection genes of the two databases (100 HNRNPC-binding proteins by STRING; 200 HNRNPC-related genes using the cBioPortal online). (**C**) The scatter plot was used to observe the correlation between KPNA4, SMARCC1, YWHAE and HNRNPC. (**D**) Corresponding heatmap data for KPNA4, SMARCC1, YWHAE in pan-cancer.

To further elucidate the molecular mechanism by which the HNRNPC gene contributes to carcinogenesis, we searched for HNRNPC-targeting proteins and HNRNPC expression-correlated genes using a variety of pathway enrichment studies. The STRING program was used to identify and validate 100 HNRNPC-binding proteins. The protein interaction network is shown in Figure 7A. The cBioPortal web server was used to retrieve all TCGA tumor expression data and the top 200 genes connected with HNRNPC expression. The associated heatmap data revealed a positive connection between HNRNPC and many genes associated with most types of cancers (Figure 7B). SMARCC1, KPNA4, and YWHAE were identified as common members of the aforementioned two groups by an intersection analysis. As seen in Figure 7C, the amount of HNRNPC expression was strongly linked with the SMARCC1 (R = 0.6), KPNA4 (R = 0.43), and YWHAE (R = 0.55) genes (all P < 0.001) (Figure 7D).

**Figure 8:**
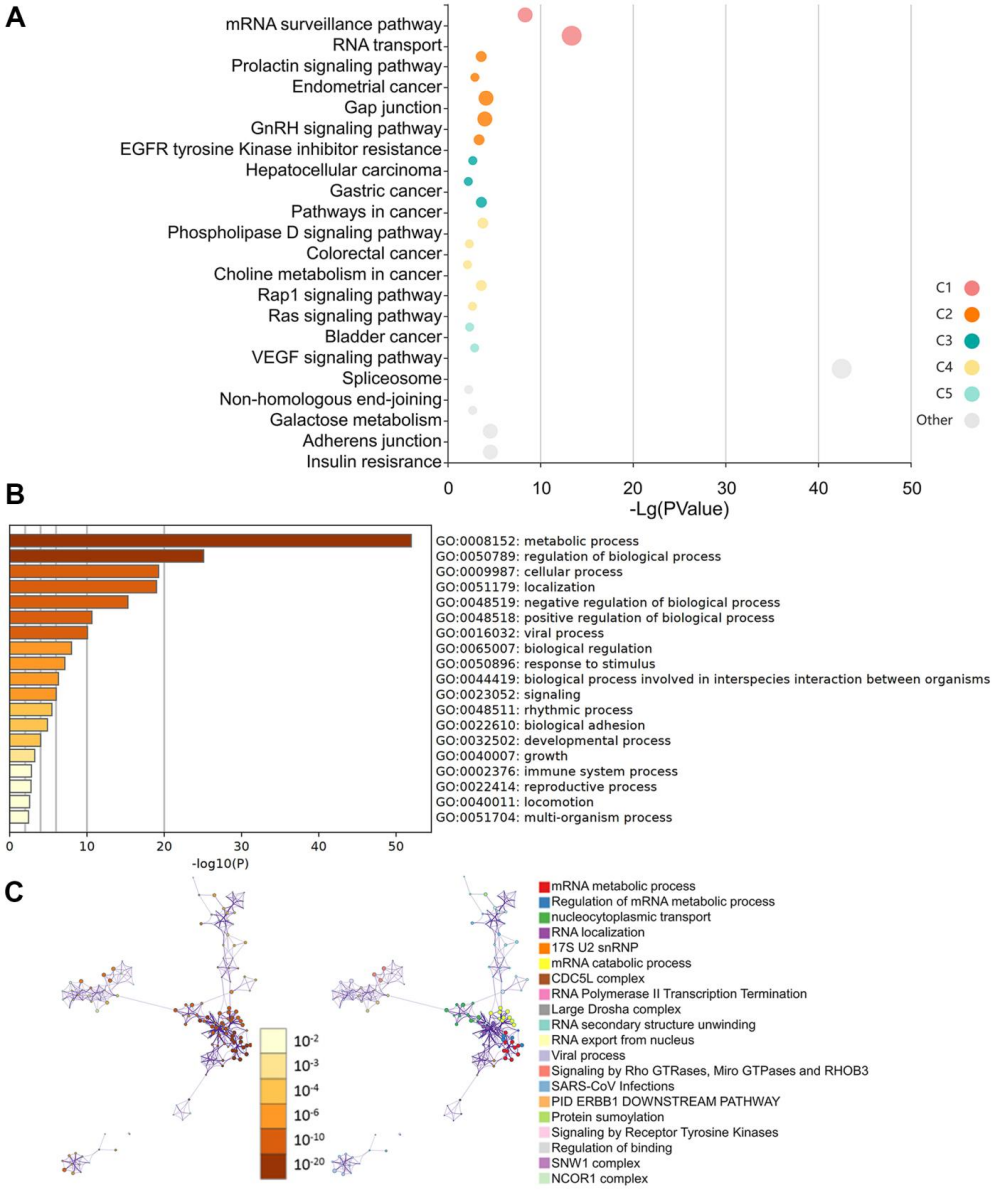
**Molecular pathways involved in HNRNPC-related genes.** (**A**) Analysis of KEGG pathways using HNRNPC-binding (STING database) and related genes (the cBioPortal online) with a bubble chart. (**B**) Bar chart illustrating the molecular function data for HNRNPC-binding (STING database) and related genes (the cBioPortal online). (**C**) Bar chart illustrating the molecular function data for HNRNPC-binding (STING database) and related genes (the cBioPortal online).

Additionally, the KEGG data indicate that the “VEGF signaling pathway” and the “RNA transport” may be implicated in the way HNRNPC influence tumor development (Figure 8A). Additionally, the GO enrichment analysis revealed that the majority of genes were engaged in metabolic and regulatory biological processes, including mRNA metabolism, nucleocytoplasmic transport, and RNA location (Figure 8B, 8C).

## DISCUSSION

HNRNPC is a critical protein that plays a role in the cell cycle, especially in transcription and translation [27]. HNRNPC was shown to be involved in the initial steps of spliceosome assembly and pre-mRNA splicing [28]. The TreeFam website (http://www.treefam.org) was used in this study to demonstrate the conservation of HNRNPC structure across many species (Supplementary Figure 1). HNRNPC was shown to have comparable roles in a variety of animals. HNRNPC expression of DNA, RNA, and protein did not vary significantly among the various tissues investigated (Figure 1A). Single cell sequencing study revealed that HNRNPC is expressed in all organ cells (Supplementary Figure 2). As a result, no significant change in HNRNPC expression was observed between tissues and cells. Additionally, it may be suggested that an aberrant HNRNPC expression was a significant factor in abnormal cell behavior. Following that, the survival curve, protein phosphorylation, immunological infiltration, and molecular process will be discussed.

### Survival prognosis curve

GEPIA2 was utilized to analyze HNRNPC expression and survival maps in lung adenocarcinoma tissues of the TCGA-LUAD project (Supplementary Figure 3).

There was a strong association between HNRNPC expression in patients with LUAD (OS:P = 0.00011; DFS: P = 0.037). Then, using the Kaplan-Meier plotter, a series of survival analyses were conducted on the GSE200014/ GSE200751/ GSE216302/ GSE227110 cohorts. Our analysis revealed strong correlations between HNRNPC expression and clinical prognosis of OS and first progression-free survival. High HNRNPC expression has been related to poor outcomes in LUAD [14, 29]. Additionally, surgical therapy for LUAD is beneficial.

Moreover, the Kaplan-Meier plotter was utilized to confirm and investigate gastric cancer (GC) (Supplementary Figure 4A, 4B). In all cohorts, GC prognosis was strongly associated with HNRNPC expression in OS and PFS. High HNRNPC levels are associated with poor OS and PFS. Chemoresistance may be promoted by overexpression of HNRNPC in parental cells [30]. Additionally, there was a link between HNRNPC expression and Her-2(−) status (Supplementary Figure 4C, 4D). According to earlier studies, the link between HNRNPC and Her-2 in GC has not significantly progressed. Patients in the GSE200751 cohort had disparate patterns in terms of ovarian cancer subtypes (Supplementary Figure 5).

It has been found that enhanced expression of Mir-744-5p directly inhibited mRNA transcription and decreased nuclear factor IX (NFIX) and HNRNPC. The HNRNPC protein was previously reported to induce apoptosis. HNRNPC has been shown to inhibit mir-21 expression and phosphorylation of AKT in previous studies [26]. Additionally, it was discovered that Taxol had a greater effect on ovarian cancers with elevated HNRNPC expression in the survival curve (Supplementary Figure 5). Taxol may act as a selective HNRNPC inhibitor.

### Phosphorylation of proteins

Using the CPTAC database, HNRNPC protein expression was found to be greater in cancerous than in normal tissues (Figure 1D). Additionally, HNRNPC contains a large number of phosphorylation sites that are statistically significant in many types of cancer (Figure 5). S220 expression was significantly different in all six malignancies. The S220 locus may operate as a phosphorylation site for HNRNPC activation in cancers. The cell cycle-regulated protein kinase has been shown to be involved in the phosphorylation of the HNRNPC protein. Phosphorylation of HNRNPC may regulate the formation and disassembly of HNRNP complexes, as well as the activity of RNA binding proteins and cell localization [31]. Meanwhile, it has been established that HNRNPC may bind to p53, inhibit its phosphorylation, and block the apoptotic pathway [32, 33]. In summary, S220 may be a key location of phosphorylation for HNRNPC proteins, and it may play a role in malignancy and cancer progression.

### Immune infiltration

HNRNPC is intimately associated with the generation of Interferon γ (IFN-γ) in tumor cells, and it may play a significant role in the tumor microenvironment due to its unique immunological activity [9, 10].

HNRNPC expression was observed to be connected with tumor-associated fibroblast expression in LUSC, TGCT, and THYM (Figure 6A, 6B). Additionally, the expression of HNRNPC and CD8+ T cells was almost perfectly associated with uveal melanoma (UVM) (Supplementary Figure 6A). Similarly, HNRNPC expression in HNSC, HNSC-HPV-, SKCM, and SKCM-Metastasis was favorably linked with CD4+ T cells (Supplementary Figure 6B). HNRNPC expression was shown to be negatively correlated with T cell regulatory gene expression in TGCT (Supplementary Figure 6C). HNRNPC expression was strongly linked with B cells in LIHC and with monocytes in SKCM (Supplementary Figure 6E, 6F). Diverse macrophage types and HNRNPC expression patterns were observed (Supplementary Figure 6G). This might be because M0, M1, and M2 cells play distinct roles in the immunological inflammatory response. In summary, HNRNPC expression and immune cell infiltration patterns vary significantly among cancer types. The intricacy and variety of the immunological activity of HNRNPC will be a strategic challenge for future cancer research.

### The molecular mechanism

In pan-cancer investigations, it was postulated for the first time that HNRNPC may be connected to the VEGF pathway (Figure 8A). Additionally, GO analysis revealed that HNRNPC's primary activities remained focused on mRNA metabolism control, nuclear cytoplasmic transport, and RNA localization (Figure 8B, 8C). In summary, the foregoing pan-cancer investigations established for the first time a statistically significant correlation between HNRNPC expression and clinical prognosis, protein phosphorylation, immune cell infiltration, and gene mutation. The subsequent examination of clinical tumor samples will provide fresh light on the role of HNRNPC in tumorigenesis.

## Supplementary Material

Supplementary Figures
