# Supplementary material for: A systematic pan-cancer study demonstrates the oncogenic function of heterogeneous nuclear ribonucleoprotein C
Source: Aging (Albany NY). 2022 Mar 28;14(6):2880–901. doi: 10.18632/aging.203981 (PMC9004556; doi:10.18632/aging.203981)
Supplement: Supplementary Figures [file aging-14-203981-s001.pdf]

SUPPLEMENTARY FIGURES

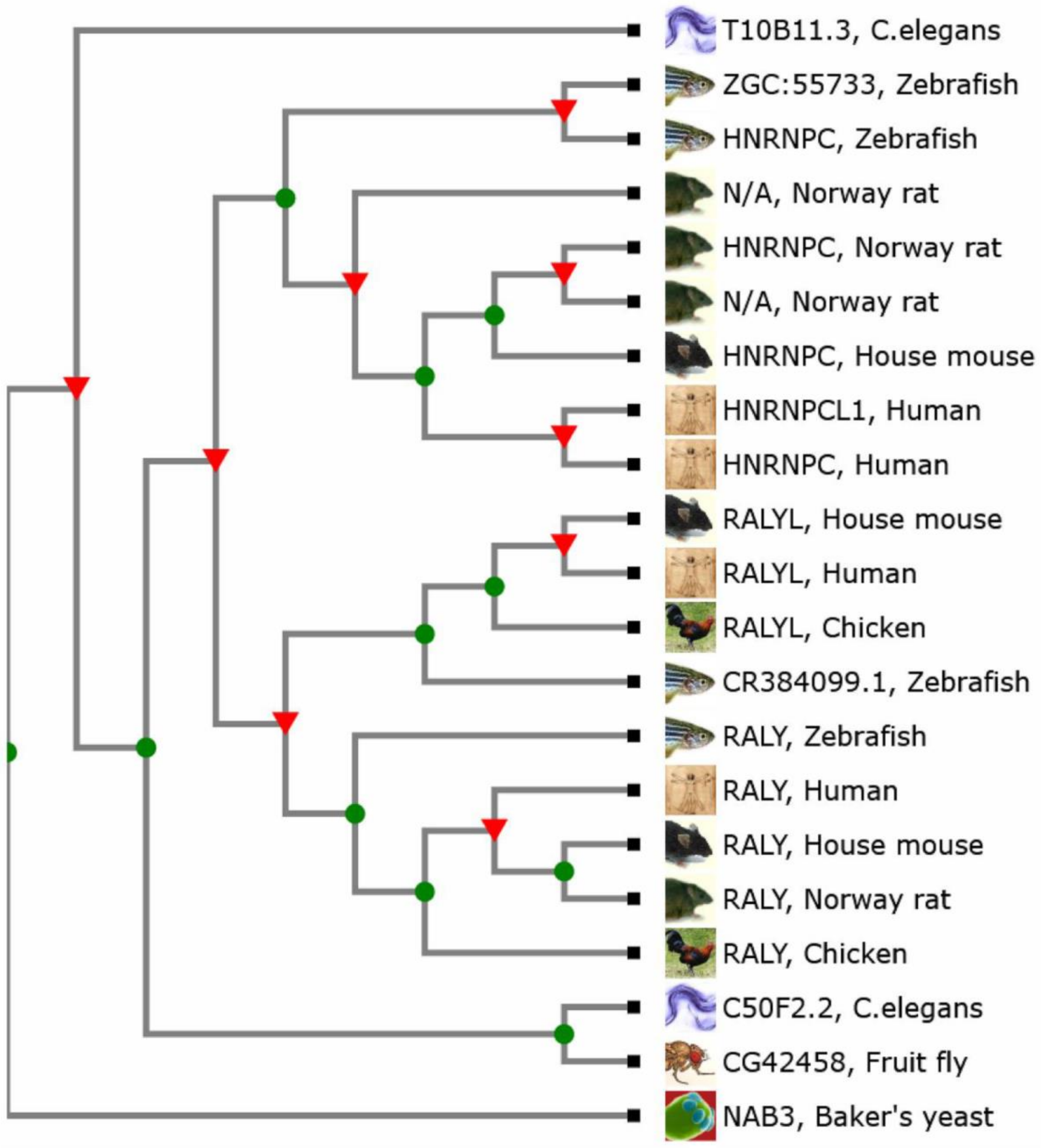

Supplementary Figure 1. Analysis the gene tree about HNRNPC in different races by TreeFam web (http://www.treefam.org).

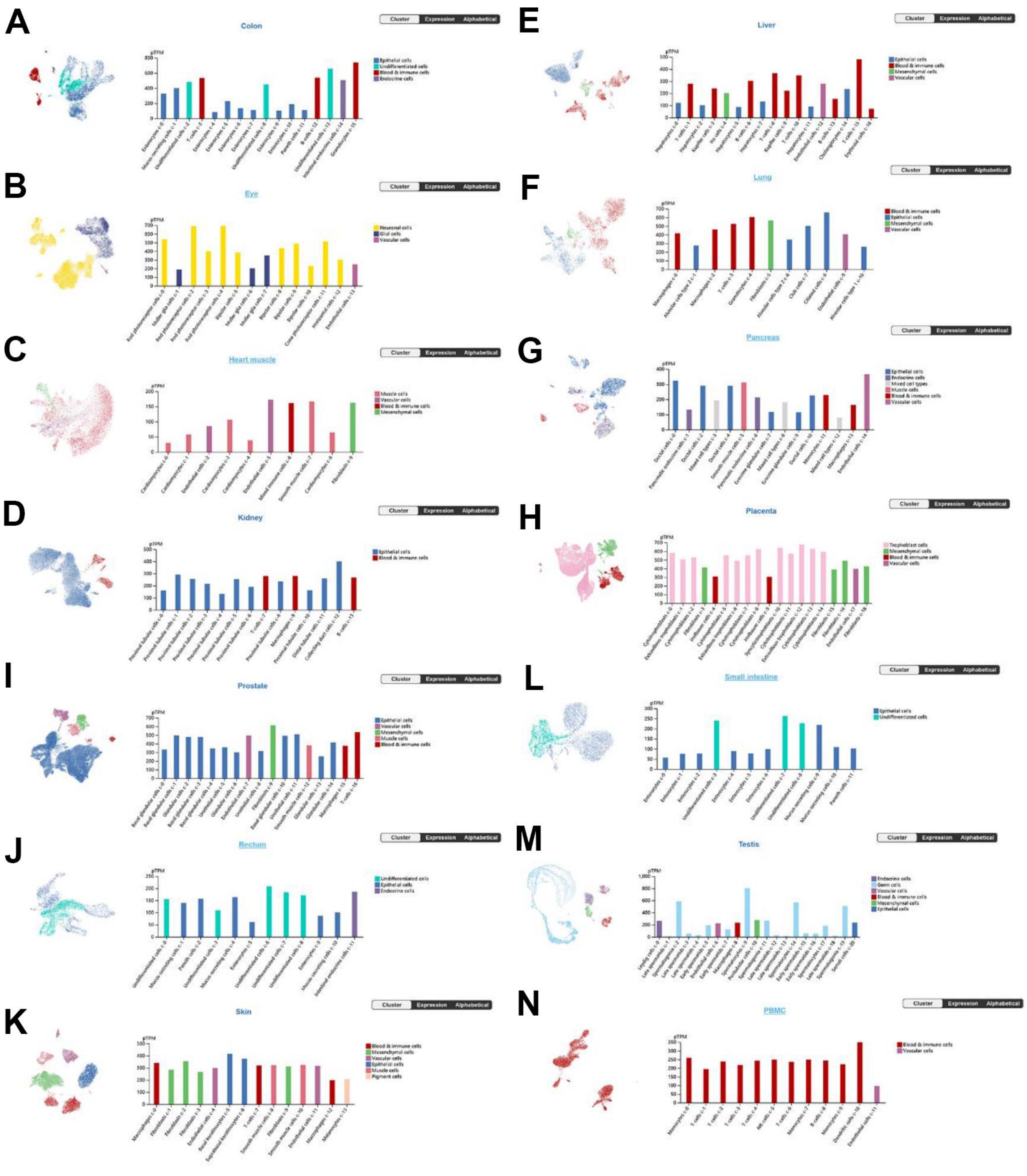

Supplementary Figure 2. Analysis the single cell types atlas in different organs by HPA database. (**A**) Colon, (**B**) Eye, (**C**) Heart muscle, (**D**) Kidney, (**E**) Liver, (**F**) Lung, (**G**) Pancreas, (**H**) Placenta, (**I**) Prostate, (**J**) Rectum, (**K**) Skin, (**L**) Small intestine, (**M**) Testis, (**N**) PBMC.

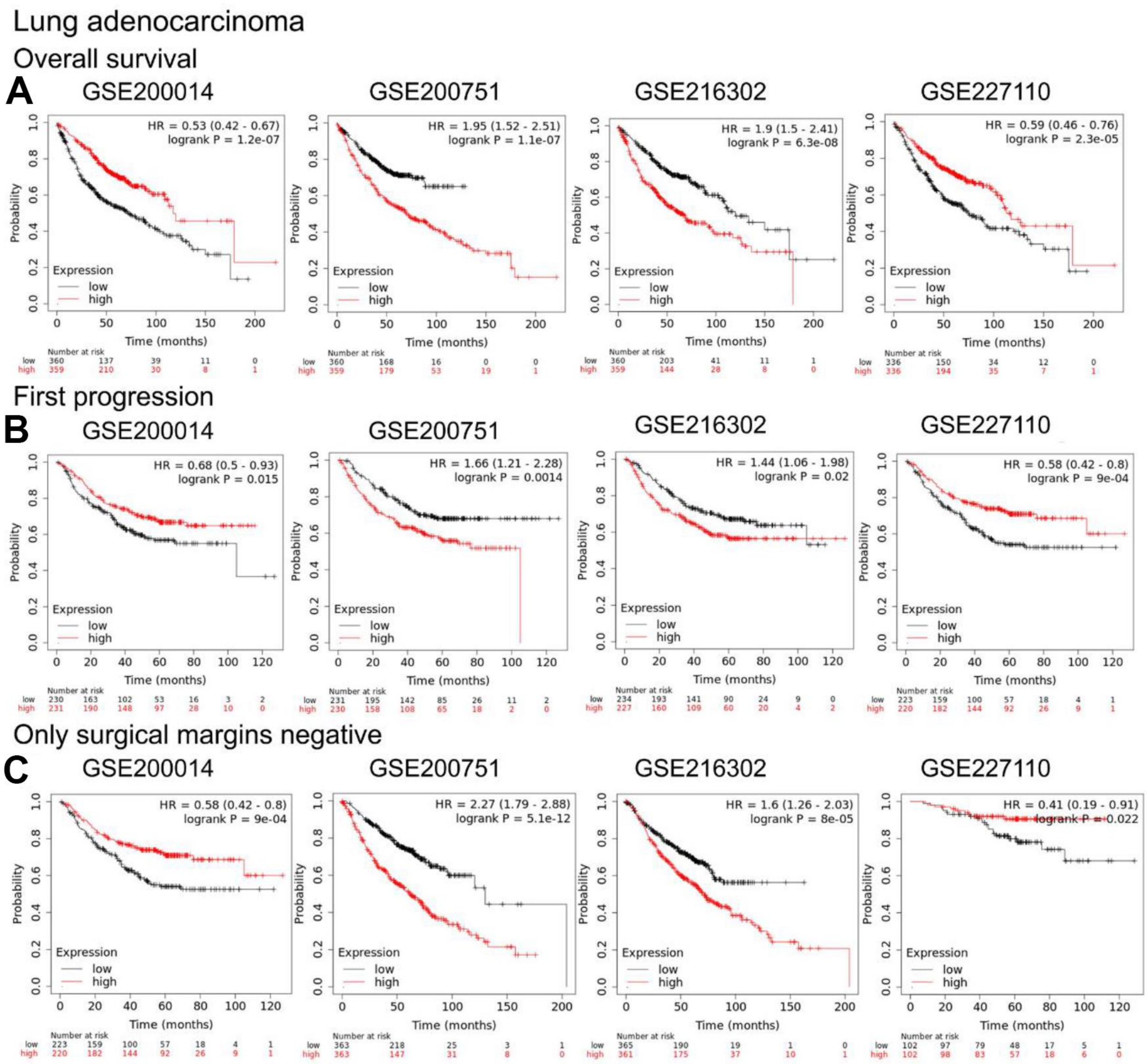

Supplementary Figure 3. Relationship between HNRNPC expression and prognosis of Lung adenocarcinoma, including (**A**) Overall survival progression, (**B**) First progression and (**C**) Only surgical margins negative progression.

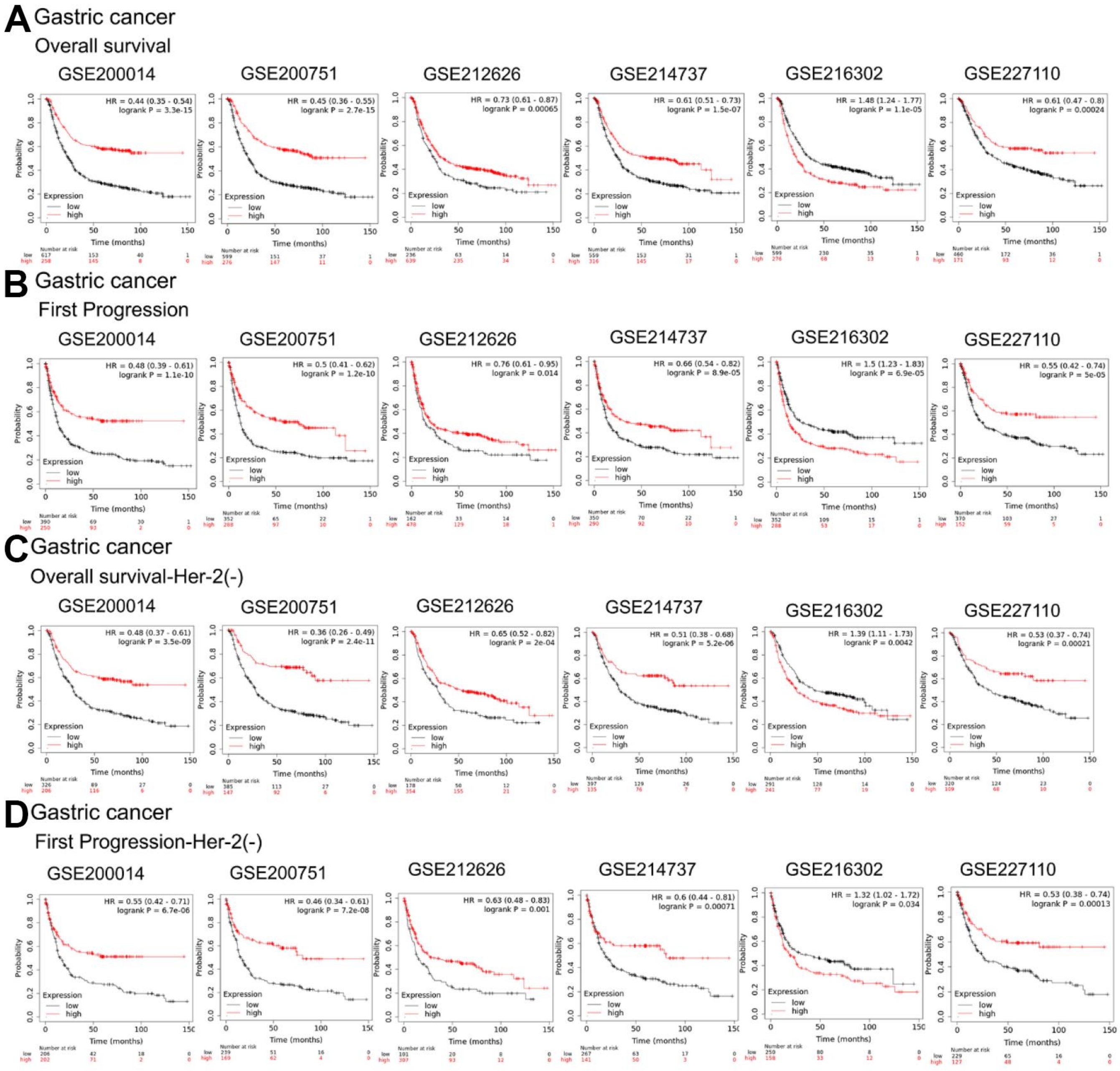

Supplementary Figure 4. Relationship between HNRNPC expression and prognosis of Gastric cancer, including (**A**) Overall survival progression, (**B**) First progression, (**C**) Overall survival progression-Her-2 and (**D**) First progression-Her-2.

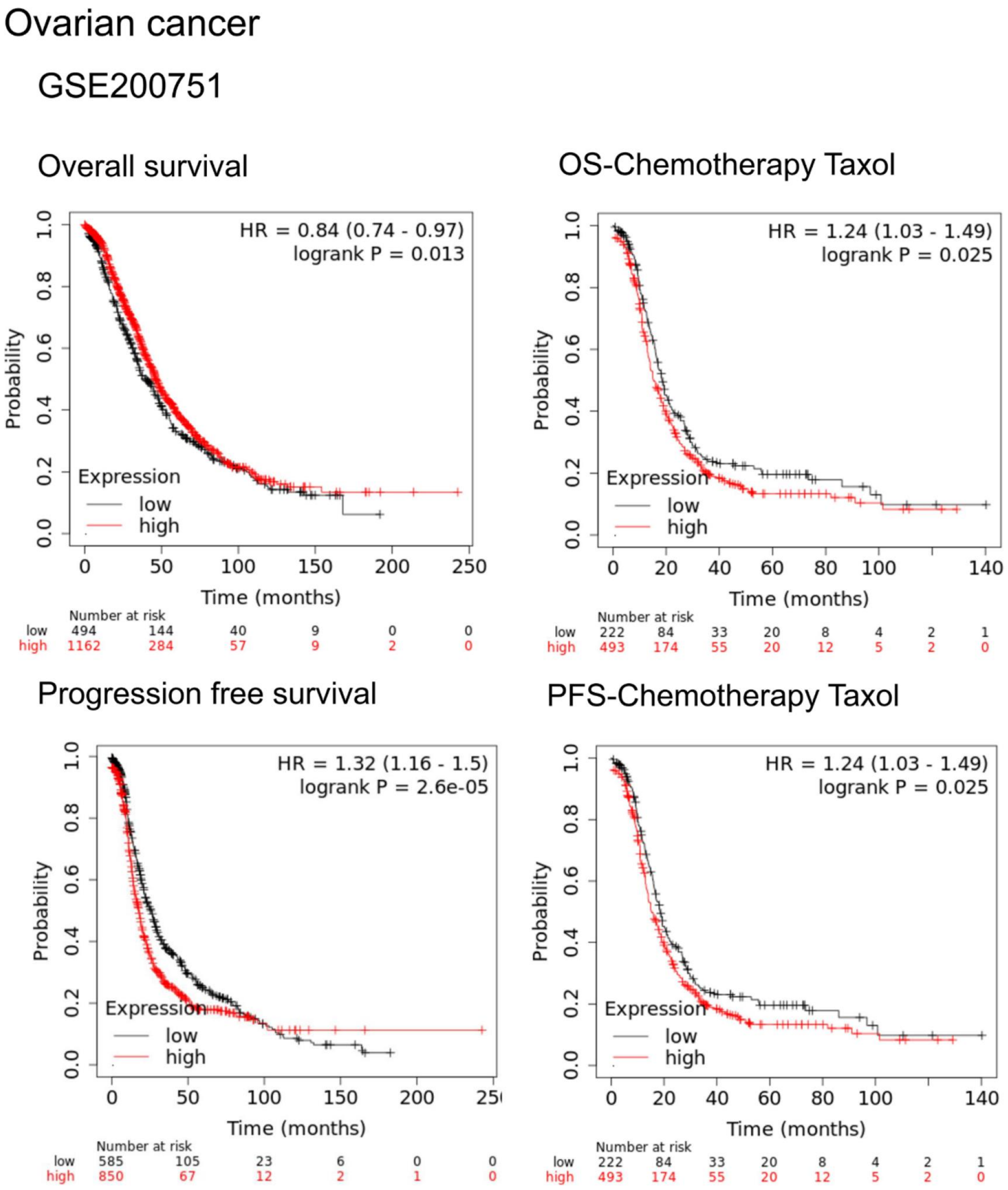

Supplementary Figure 5. Relationship between HNRNPC expression and prognosis of ovarian cancer.

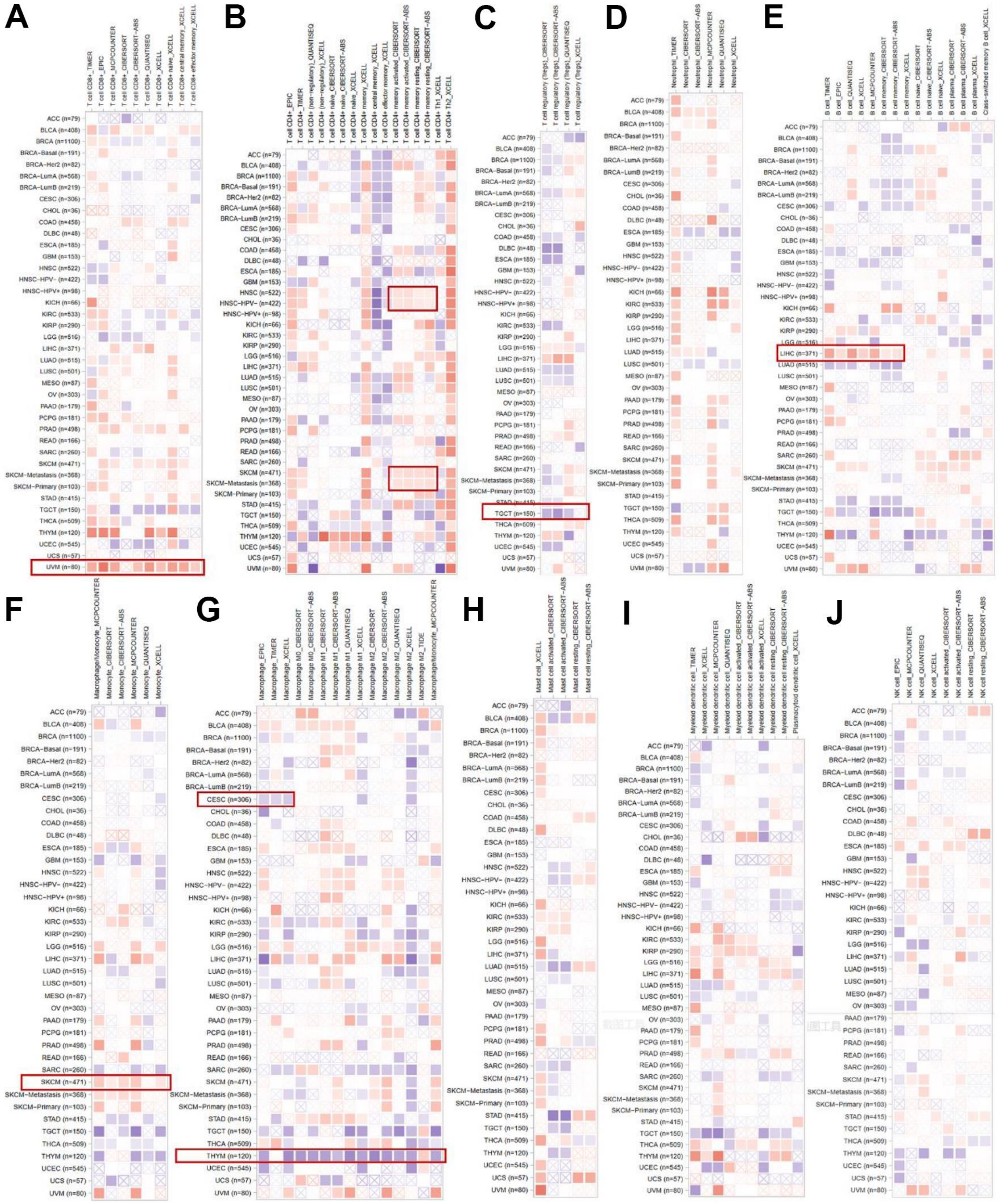

Supplementary Figure 6. Some algorithms were used to explore the potential correlation between immune cells and cancer in TCGA, such as (**A**) CD8+ T cell, (**B**) CD4+ T cell, (**C**) regulatory T cell, (**D**) Neutrophil, (**E**) B cell, (**F**) Monocyte, (**G**) Macrophage, (**H**) Mast cell, (**I**) Myeloid dendritic cell, (**J**) NK cell.
